# Psychometric Evaluation of the Canadian Nurse Informatics Competency Assessment Scale and the Digital-Technology Self-Efficacy Scale Among Saudi Nursing Students: Cross-Sectional Study

**DOI:** 10.2196/88075

**Published:** 2026-05-05

**Authors:** Nader Alnomasy, Habib Alrashedi, Sharifah Alsayed, Petelyne Pangket, Ebtsam Abou Hashish, Razan Alsayed, Romeo Jr Mostoles

**Affiliations:** 1 Medical Surgical Nursing Department, College of Nursing, University of Ha'il, 3871, Hail, Ha'il Region, 55349, Saudi Arabia, 966 532476596, 966 532476596; 2 Medical Surgical Nursing Department, University of Ha'il, Hail, Ha'il Region, Saudi Arabia; 3 College of Nursing, King Saud bin Abdulaziz University for Health Sciences, Jeddah, Mecca Region, Saudi Arabia; 4 Medical Surgical Department, College of Nursing, Taif University, Taif, Mecca Region, Saudi Arabia; 5 King Abdullah International Medical Research Center, Jeddah, Mecca Region, Saudi Arabia; 6 Ministry of National Guard Health Affairs, Jeddah, Mecca Region, Saudi Arabia; 7 Faculty of Nursing, Alexandria University, Alexandria, Egypt; 8 Nursing Education Department, King Fahd Armed Forces Hospital, Jeddah, Mecca Region, Saudi Arabia; 9 Department of Psychiatric and Mental Health Nursing, College of Nursing, University of Ha'il, Hail, Ha'il Region, Saudi Arabia

**Keywords:** nursing informatics competency, digital self-efficacy, nursing students, Saudi Vision 2030, health care digital transformation

## Abstract

**Background:**

The integration of digital health technologies into nursing education in Saudi Arabia requires reliable tools to assess nursing informatics competency and digital technology self-efficacy among students.

**Objective:**

This study aimed to evaluate the reliability and validity of the Canadian Nursing Informatics Competency Assessment Scale (C-NICAS) and Digital Technology Self-Efficacy (DT-SE) scale among undergraduate nursing students at a Saudi university.

**Methods:**

A descriptive cross-sectional survey of 243 undergraduate nursing students at the University of Ha’il was conducted using the C-NICAS and DT-SE. Internal consistency was examined using Cronbach α, and construct validity was assessed using exploratory and confirmatory factor analyses.

**Results:**

A total of 243 students participated (mean C-NICAS score 54.0, SD 16.9; mean DT-SE score 2.7, SD 0.56). Both scales showed good internal consistency (C-NICAS total α=0.90; DT-SE α=0.80). C-NICAS demonstrated a multidimensional factor structure with an acceptable model fit (comparative fit index=1.00; root mean square error of approximation=0.081), whereas DT-SE showed a 3-factor structure with a suboptimal confirmatory model fit (comparative fit index=0.76, root mean square error of approximation=0.146).

**Conclusions:**

The C-NICAS and the DT-SE are suitable for assessing informatics competency and digital self-efficacy among undergraduate nursing students at this institution, although further refinement of the DT-SE may improve model fit. These validated tools can inform curriculum reform at this and similar institutions in Saudi Arabia and support the digital health goals of Saudi Vision 2030.

## Introduction

Health care providers worldwide are urgently challenged to integrate digital health technologies into their practices, requiring them to acquire new competencies, especially nurses, the largest single profession in health care systems [1,2]. Electronic health records (EHRs), decision support systems, and mobile health apps are increasingly used to provide safe, efficient, and patient-centered care [3]. Informatics competencies are essential professional requirements for all practicing nurses today and have been established through international initiatives, including the Technology Informatics Guiding Education Reform (TIGER) initiative. TIGER seeks to prepare new nursing graduates to be digitally literate to enhance evidence-based practice and collaboration among health care disciplines [4]. Additionally, the International Medical Informatics Association (IMIA) has published global educational recommendations that define core biomedical and health informatics competencies and provide a framework for designing and accrediting health and nursing informatics curricula across different professional roles and levels of specialization [5,6]. In parallel, the World Health Organization’s *Global Strategy on Digital Health 2020–2025* [7] emphasizes that strengthening the digital competencies of the health workforce, including nurses, through pre-service and in-service education is a key component of achieving safe, effective, and equitable digital health implementation. The World Health Organization guidance on digital education further highlights that integrating digital health content and e-learning into health professional curricula is essential for building workforce capacity and supporting lifelong learning in rapidly evolving digital health systems [8].

Saudi Vision 2030 represents Saudi Arabia’s national vision for economic development and social reform, with a focus on digital transformation and the adoption of eHealth as a mechanism to cultivate a modernized and technologically prepared workforce to address future digital challenges in health care [9]. Investments in national EHR and health information systems demonstrate this commitment to digital transformation [10]. Although significant progress has been made toward achieving the digital transformation of health care services in Saudi Arabia, barriers continue to exist among nursing students; newly graduated nurses from Saudi Arabia continue to indicate that they possess modest levels of informatics competency and low confidence in their ability to apply digital technology in both academic and clinical settings [2,11]. The results of these studies clearly illustrate an important disconnect between the national goal of a fully developed digital health care system and the preparedness of the future nursing workforce to deliver services in this system [12].

Several international studies support the use of the Canadian Nursing Informatics Competency Assessment Scale (C-NICAS) in both high- and low-income countries. Internationally, studies validating the tool have provided evidence for its reliability and validity across multiple nursing populations in Canada [13,14] and have demonstrated high levels of reliability during adaptation efforts in Australia [15]. The Digital Technology Self-Efficacy (DT-SE) scale, which measures self-efficacy in relation to digital technology, has been used in Europe, Asia, and the Arab world. These studies have demonstrated consistent reliability across regions [16,17]. Systematic review results show that when these tools are transferred to other countries, they do not always perform similarly in terms of psychometric properties. Therefore, it is essential to conduct local validation to account for variations in nursing education and practice settings.

However, no prior study has rigorously validated the C-NICAS and DT-SE scales for Saudi undergraduate nursing students, which represents a significant gap in the field. The lack of culturally adapted and psychometrically sound measures for assessing digital competency in undergraduate nursing students in Saudi Arabia does not allow educators to reliably measure baseline digital competency, assess the effectiveness of their curricula, or make evidence-based decisions for reform, as required by the digital health priorities of Saudi Vision 2030.

Therefore, this study aimed to evaluate the reliability and validity of the C-NICAS and DT-SE scales among undergraduate nursing students in Saudi Arabia to provide robust, locally validated measures of informatics competency and digital technology self-efficacy.

This study also enables a reliable measurement of students’ digital readiness, which bridges the gap between knowledge and practice, supports the design of educational interventions, and provides benchmarks for educational institutions. Finally, it offers practical insights into developing a digitally competent nursing workforce and advancing cross-cultural scholarship in nursing informatics for nurse educators, curriculum developers, and policymakers. The validation of the C-NICAS and DT-SE tools in Saudi Arabia will further facilitate the ongoing evaluation of curriculum changes in digital health education through repeated assessments and targeted integration of simulation learning and hands-on experience using electronic medical records while supporting investment in digital resources and informing workforce modernization strategies for national health care transformation.

## Methods

### Study Design

This descriptive cross-sectional study evaluated the psychometric properties of the C-NICAS and DT-SE scale among Saudi undergraduate nursing students.

### Setting, Participants, and Sampling

Students attending the College of Nursing at the University of Ha’il were recruited between February and June 2025 for the study. A priori power calculation was conducted to identify how many participants would be needed to support a one-way ANOVA, using G*Power software (version 3.1.9.7) [18] with a small-to-medium effect size (*f*=0.25), an α level of .05, and a statistical power of 0.80. Two hundred students were required to achieve these parameters.

The most practical and efficient method for recruiting a sample within the confines of the project timeline and budget was convenience sampling. This sampling strategy allowed for the recruitment of students across multiple academic years/levels and facilitated a broader representation of student participation. However, the use of convenience sampling also introduced a risk of selection bias, in that students who were more readily available and/or engaged may have been overrepresented. Additionally, limiting recruitment to a single institution restricted the generalizability of the findings, as they may not have accurately reflected the larger population of Saudi nursing students in the country. Therefore, future studies should use multi-institutional or probability-based sampling designs to increase the representativeness and external validity of their findings.

The inclusion criteria were as follows: active students in the nursing program who were aged 18 years or older, were fluent in English, and provided informed consent. Students in their preparatory year, non-nursing majors, students on leave, those who withdrew, and visiting students not affiliated with the college were excluded. An additional 20% of the originally calculated number of participants was included to allow for attrition and missing data in participant responses.

### Rationale for Instrument Versions

Two valid instruments were used in this study. The first was C-NICAS version 2, the most recent peer-reviewed version of an instrument that represents the current informatics standards accepted throughout international competency frameworks. Research has supported the reliability and relevance of the domains of this instrument for measuring nursing informatics competency worldwide; therefore, it was determined to be appropriate for establishing benchmarks in nursing informatics competencies for undergraduate nursing education in Saudi Arabia. The second instrument was the DT-SE scale [19], which has been shown to have high psychometric properties (α>.90) and has been successfully adapted in several cultures. This scale can measure and compare self-efficacy for digital health tasks among nursing students with some degree of reliability and comparability. Both instruments provided evidence-based and internationally comparable assessments while providing locally validated measures for use within the Saudi context.

### Survey Instrument

The survey tool was divided into two components: part 1 contained demographic questions regarding age, gender, level of education, prior experience with EHRs, and individual self-perception of digital readiness. Part 2 contained the C-NICAS version 2 (4 subscales with 26 items), a tool designed to measure competency in 4 areas: foundational computer skills, information and knowledge management, professional and regulatory accountability, and use of computers in patient care. The DT-SE (17 items) measures individual self-efficacy for completing digital health tasks (Likert scale: 1=“strongly disagree” to 4=“strongly agree”; some items were reverse scored); higher scores represent greater digital self-efficacy.

### Data Collection

Data were collected from the participants using anonymous online surveys administered through a secure web-based platform. Withdrawal from the study was permissible at any time . All items were completed in a self-report format, and the participants submitted their responses electronically upon completing the survey.

### Data Analysis

Following data collection, all responses were exported to SPSS Statistics for Windows (version 29; IBM Corp) for data cleaning and analysis. Descriptive statistics (mean, median, SD, and frequency distribution) were computed to summarize the sociodemographic characteristics and overall scores on the C-NICAS and DT-SE. Reliability analyses were conducted to estimate the internal consistency coefficients for each scale and subscale, and exploratory and confirmatory factor analyses were performed to evaluate the underlying factor structures and model fit of the C-NICAS and DT-SE. Mean imputation was applied to address missing values for continuous variables, whereas mode imputation was used for missing categorical variables. Python was used to generate supplementary data visualizations to support the interpretation and presentation of the findings.

### Ethical Considerations

This study was approved by the Institutional Review Board of the University of Ha’il (approval H-2024‐437). Online informed consent was obtained from each participant before the survey was initiated. The responses were anonymous and stored in a secure location on a password-protected server that only the research team could access. Participation in the study was voluntary and did not result in any penalties. No incentives were provided for participation.

## Results

The demographic information of the 243 Saudi nursing undergraduates is presented in Table 1. Of the 243 participants, 110 (45.5%) were aged 20 years or less, 116 (47.7%) were aged between 21 and 25 years, and 17 (7%) were aged 26 years or more. The majority of participants were women (159/243, 65.4%) and a minority were men (84/243, 34.6%). There were 69 (28.4%) first-year students, 72 (29.6%) second-year students, 39 (16%) third-year students, and 63 (25.9%) fourth-year students. Of the 243 participants, 124 (51%) reported that they had completed some type of informatics-related education prior to participating in this study, and 119 (49%) stated that they had not completed any type of education related to informatics.

**Table 1.** Sociodemographic characteristics and experience/attitudes regarding technology among respondents (N=243).

		Participants, n (%)
Age		
	≤20 years	110 (45.5)
	21‐25 years	116 (47.7)
	≥26 years	17 (7.0)
Gender		
	Male	84 (34.6)
	Female	159 (65.4)
Year		
	First year	69 (28.4)
	Second year	72 (29.6)
	Third year	39 (16.0)
	Fourth year	63 (25.9)
Informatics training		
	No	119 (49.0)
	Yes	124 (51.0)
Comfort with digital technology		
	Very uncomfortable	47 (19.3)
	Uncomfortable	53 (21.8)
	Neutral	54 (22.2)
	Comfortable	89 (36.6)
Electronic health record exposure		
	No experience	72 (29.6)
	Occasional	98 (40.3)
	Frequent	73 (30.0)
PC/laptop access		
	No	49 (20.2)
	Yes	194 (79.8)
Technology for education		
	Monthly	11 (4.5)
	Weekly	32 (13.2)
	Daily	200 (82.3)
Home internet		
	Fair	19 (8.7)
	Good	66 (27.2)
	Excellent	158 (65.0)
Career area		
	Clinical care	130 (53.5)
	Academic/teaching	35 (14.4)
	Public health/community	17 (7.0)
	Administration/leadership	14 (5.8)
	Informatics	14 (5.8)
	Undecided	33 (13.6)

Of the 243 participants, 47 (19.3%) stated that they felt very uncomfortable using digital technology, 53 (21.8%) felt uncomfortable, 54 (22.2%) felt neutral about using digital technology, and 89 (36.6%) felt comfortable. Of the participants, 72 (29.6%) stated that they had never seen an EHR before, 98 (40.3%) reported seeing EHRs monthly or less, and 73 (30%) reported using EHRs every week or more.

A large proportion of participants (194/243, 79.8%) either owned their own computers/laptops or had regular access to them. However, 49 (20.2%) participants indicated that they did not have regular access to a personal computer/laptop. Regarding the use of technology for educational purposes, 11 (4.5%) reported that they used this technology monthly, 32 (13.2%) used it weekly, and 200 (82.3%) reported that they used it daily.

In terms of accessing the internet at home, 19 (8.7%) of the participants rated their access as fair, 66 (27.2%) rated their access as good, and 158 (65%) rated their access as excellent. When asked what areas of nursing they planned to pursue after graduation, 130 (53.5%) of the participants planned to pursue roles in direct patient care in hospitals or clinics, 35 (14.4%) wanted to pursue roles in academia or as educators, 17 (7%) wanted to pursue roles in public health/community nursing, 14 (5.8%) wanted to pursue administrative/leadership roles, 14 (5.8%) were interested in pursuing roles in nursing informatics, and 33 (13.6%) were undecided about what area of nursing they would pursue.

Table 2 presents descriptive statistics for the C-NICAS and DT-SE scales. Mean scores across the C-NICAS domains were relatively consistent, including information and communication technology (ICT) skills (mean 2.6, SD 0.93), knowledge (mean 2.5, SD 0.87), accountability (mean 2.6, SD 0.89), and use (mean 2.6, SD 0.85). The overall mean score for the C-NICAS was 54 (SD 16.9), suggesting a moderate level of informatics competency among the students. The mean DT-SE score was 2.7 (SD 0.56), indicating moderate self-efficacy in using digital technology.

**Table 2.** Descriptive statistics of informatics competency and digital self-efficacy.

Variable	Scores, mean (SD)	
Foundational information and communication technology skills	2.6 (0.93)	
Information and knowledge management	2.5 (0.87)	
Professional and regulatory accountability	2.6 (0.89)	
Use of information and communication technology in patient care	2.6 (0.85)	
Overall C-NICAS^a^	54 (16.9)	
DT-SE^b^	2.7 (0.56)	

a Canadian Nursing Informatics Competency Assessment Scale.

b Digital Technology Self-Efficacy.

Table 3 summarizes the reliability testing results for the C-NICAS. Cronbach α values indicated good to excellent internal consistency for all domains: foundational ICT skills (α=0.70), information and knowledge management (α=0.80), professional and regulatory accountability (α=0.82), and use of ICT in patient care (α=0*.*90). The overall the C-NICAS demonstrated excellent reliability (α=0.90). The DT-SE scale showed good reliability (α=0.80).

**Table 3.** Internal consistency reliability of the Canadian Nursing Informatics Competency Assessment Scale (C-NICAS) and Digital Technology Self-Efficacy (DT-SE) scale.

Scale	Items, n	Cronbach α	Level of internal consistency
Foundational information and communication technology skills	2	0.70	Good
Information and knowledge management	6	0.80	Good
Professional and regulatory accountability	6	0.82	Good
Use of information and communication technology in patient care	10	0.90	Excellent
C-NICAS	26	0.90	Excellent
DT-SE	17	0.80	Very good

### Factor Structure of C-NICAS

Exploratory factor analysis supported a multidimensional structure for the C-NICAS, with item commonalities in an acceptable range and excellent sampling adequacy (Kaiser-Meyer-Olkin=0.976). Confirmatory factor analysis indicated an acceptable model fit for the 4-factor model (comparative fit index=1.000; incremental fit index=1.000; root mean square error of approximation=0.081; *χ*²_1_=2.02). The full factor loading matrix and related statistics are presented in Table 1 in Multimedia Appendix 1, with the corresponding factor loading plot in Figure 1 in Multimedia Appendix 1 and detailed CFA fit indices in Table 2 in Multimedia Appendix 1.

### Factor Structure of DT-SE

For the DT-SE, sampling adequacy was strong (Kaiser-Meyer-Olkin=0.930), and 3 factors emerged in the exploratory factor analysis, explaining 67.7% of the total variance. The rotated factor loadings and variance explained are shown in Tables 3 and 4 in Multimedia Appendix 1, and the factor-loading plot is shown in Figure 2 in Multimedia Appendix 1. Confirmatory factor analysis of the DT-SE indicated a suboptimal model fit (*χ*²_119_=877.10; comparative fit index=0.76; root mean square error of approximation=0.146), suggesting the need for further refinement of the scale in this context. The detailed CFA indices are summarized in Tables 5 and 6 and Figure 3 in Multimedia Appendix 1.

## Discussion

In this cross-sectional study, the C-NICAS demonstrated excellent internal consistency and a stable multidimensional factor structure, confirming its suitability for assessing informatics competency among undergraduate nursing students at this Saudi institution. The DT-SE scale showed good internal consistency and an interpretable factor structure, although some model fit indices indicated that further refinement may enhance its performance. Overall, the participants reported moderate levels of informatics competency and digital technology self-efficacy, with stronger foundational ICT skills than advanced digital readiness.

### Psychometric Properties of C-NICAS and DT-SE

This reliability/validity assessment found that the C-NICAS [20] and DT-SE scale were both highly reliable and valid and thus appropriate for assessing informatics competency and self-efficacy in an educational setting of this type. The strong psychometric properties of both scales indicate that items within each scale group appropriately represent the theoretically supported domains and thus provide a meaningful assessment of competency and self-efficacy among undergraduate nursing students at the University of Ha’il in Saudi Arabia.

The results support similar validation assessments in Canada [21] and demonstrate that the C-NICAS has excellent internal consistency and a valid factor structure across multiple cohorts of undergraduate nursing students. Another similar validation assessment [22] for the Arabic version of the Self-Assessment Nursing Informatics Competencies Scale, which showed high reliability when used to assess nursing informatics competency among Arab nursing students, further supports the use of these measures to assess informatics competency in the Saudi educational context. Together, these studies demonstrate that valid and reliable scales are important for measuring nursing informatics competency.

Conte et al [23] pointed out that there is a possibility of reduced reliability when adapting self-efficacy scales to different cultures. Furthermore, a systematic review of the literature by Al-Qudah et al [24] concluded that nursing informatics scales exhibit variable reliability and validity, depending on the specific population and setting. Both research groups agreed that these scales must be refined and validated over time if they are to be used effectively on an international scale.

Validated and reliable measures of nursing informatics competencies and self-efficacy will provide Saudi nursing educators with data to assess students’ readiness to learn, identify strong and weak areas, and provide an evidence base for changes in nursing education programs. The use of valid tools also supports continuing education and contributes to the transformation of the Saudi Arabian health care system by linking nurses’ skills to the national digital health objectives.

Although the scales have strengths, their two main limitations are generalizability and fit for culture (especially for the DT-SE). Studies using larger samples and multiple iterations of the scales would assist in further refining the tools, following the authors’ recommendations, to ensure continued utility and reliability as standards for health care and informatics continue to evolve in the Kingdom of Saudi Arabia.

### Informatics Competency and Digital Self-Efficacy Levels

This study indicates that the undergraduate nursing students at this institution have an average level of informatics competence and a moderate amount of digital self-confidence; thus, they have partially adopted the digital requirements of today’s health care environment but demonstrate a gap between what they are taught about clinical informatics and how they apply it in the workplace. Students have digital skills and confidence in performing these tasks; however, their ability to apply and integrate these skills into more complex applications is less developed than their basic skills.

Compared with their global counterparts, nurses in Jordan demonstrated an almost identical profile of moderate-to-high levels of informatics competency as the participants in this study, including strong performance in general basic computer skills and much weaker performance in clinical informatics subdomain areas [25]. A similar pattern has been observed in Australia and several other Arab countries, as well as in many other parts of the world, where nursing students have shown evidence of being competent in routine digital use; however, they are often found to be lacking in specialized areas such as system management and decision-making [26-29]. The similarities among these findings support the notion that this issue is universal in nature.

Recent studies have also reported similar patterns in nursing informatics education. One recent study found that nursing students typically show higher competence in basic computer skills than in clinical information management and decision support tasks [6]. A further study proposed an undergraduate informatics blueprint that stressed progressive development from foundational to advanced competencies and explicit alignment with IMIA and TIGER recommendations, supporting the need for structured, longitudinal integration of informatics in nursing programs [29].

The level of self-confidence exhibited by nurses regarding digital abilities was similarly modest and reflects current global trends, where confidence in using technology for everyday tasks is commonplace, yet proficiency is limited in advanced applications of informatics [13,30]. While early exposure to digital tools, along with some education, support, and resources, has contributed to moderate progress in improving digital self-efficacy among Jordanian nursing students, limitations remain owing to the lack of preparation in advanced informatics, varied faculty expertise, and inconsistency in the availability of adequate technology and digital systems to support digital readiness [3]. Generational changes and increased digital exposure are likely to improve the basic competencies of nursing students; however, instructional and systemic barriers persist.

The data indicate that nursing programs in Saudi Arabia, including the program at this institution, have made some advancements in digital health; however, further strategic investments are needed to fully support the curriculum design, faculty development, and experiential learning necessary to meet the needs of clinical practice. The use of validated assessments, simulations using artificial intelligence technology, and increased emphasis on interprofessional informatics education will be the key next steps [4,31]. Ultimately, while moderate competency and self-efficacy levels suggest an advancement toward meeting the goals of Saudi Vision 2030, the overall implication of this study is that there continues to be a need for strategic reforms at all educational levels to ensure that graduates can assume leadership roles in future digitally advanced health care systems.

### Implications for Nursing Education and Curriculum Development

This research has provided many critical findings that are relevant to nursing education, nursing program assessment, and curriculum development here and potentially elsewhere in Saudi Arabia, in support of Saudi Vision 2030 and global digital transformation objectives in health care.

First, because the nursing students in this sample possessed average levels of informatics competence and digital self-efficacy, there is an imperative for nursing curricula to enhance students’ learning experiences with respect to digital health and informatics. For example, rather than simply exposing students to digital health as part of their introductory courses, nursing programs should systematically integrate advanced informatics education, simulation-based learning, EHR training, and education on artificial intelligence and decision support systems into the 4-year undergraduate nursing education experience. By doing so, students will be able to progress from a beginner level of digital literacy to being capable of effectively using emerging technologies in the delivery of patient care.

Second, by using these assessments (the DT-SE and C-NICAS) systematically, educators will be empowered to use data-driven insights when making decisions about the ongoing evaluation of nursing programs and curriculum enhancements. Ongoing assessment of nursing students’ informatics competence will empower educators to assess potential weaknesses in their students’ abilities to engage in clinical practice using digital technologies, adapt to new clinical requirements, and modify the content of their curricula to be consistent with national and international priorities related to digital health.

Additionally, these enhancements will directly contribute to the goals of Saudi Vision 2030, specifically by providing a future-ready nursing workforce that can assist in promoting and implementing advancements in health care through engagement in national eHealth initiatives and the provision of technology-enabled patient care. Furthermore, to ensure the sustainability of the positive outcomes of this study and to continue to use best practices in both educational and clinical environments, it is important for faculty to continuously receive professional development and for the institution to invest in its digital infrastructure.

Finally, the results of this study further emphasize the importance of innovation in curriculum design and competency-based education in developing nurses with the ability to meet the challenges associated with the digital transformation of health systems globally. Through the alignment of nursing education in Saudi Arabia (particularly in institutions similar to the one in which this study was conducted) with these principles, the health care system will be able to develop professionals who can deliver high-quality, safe, technology-enhanced care to patients.

### Study Limitations

This study had several important limitations. The generalizability of the findings is limited by the use of a convenience sample recruited from only one university, and the results should therefore be interpreted as reflecting the students at this institution rather than all Saudi nursing students. Since participants were asked to report their own behavior, there is a potential for both recall and social desirability biases due to the self-reporting nature of the items. Furthermore, the cross-sectional nature of the research design precludes making causal statements about the relationships observed between variables, and possible scale adaptation issues may limit the accuracy of the scales in representing the context being studied. There is a possibility that non-response (dropout) bias may have affected the findings as well, because less technologically savvy students may not have been represented.

### Recommendations

To address the gaps identified in this study, we recommend that curriculum developers continue to develop and implement advanced informatics content, simulation-based learning, and practical EHR training across undergraduate nursing programs at this and other institutions in Saudi Arabia. In addition, opportunities for faculty development and long-term investments in digital infrastructure are required to support and enhance the digital health competencies of nursing faculty members. Policymakers should also provide incentives for the continued implementation and development of standardized and psychometrically sound assessment tools, such as the C-NICAS and DT-SE, for the ongoing monitoring and evaluation of curricula at both the institutional and national levels. We believe that this will help Saudi nursing graduates in an increasingly digitalized health care environment.

Future studies should use multi-institutional, longitudinal research designs and incorporate culturally appropriate tools to increase the representativeness of findings and enable follow-up assessments of curricular impact over time. Future research should also include refinement of the DT-SE scale to improve model fit and identify targeted interventions (eg, simulation and AI-based informatics training) to address existing gaps in advanced digital preparedness among nursing students. Future research examining the association between nursing students’ informatics competency and patient outcomes will help optimize nursing education to meet national and international priorities.

### Conclusion

The validation of assessments that measure digital competency and self-efficacy is necessary to enhance nursing education in Saudi Arabia. This descriptive cross-sectional study administered the C-NICAS and DT-SE to undergraduate nursing students at a Saudi university, finding that both scales demonstrated strong psychometric properties. The results indicated that nursing students displayed moderate levels of informatics competency and digital technology self-efficacy; specifically, they demonstrated adequate basic-level skills but lacked advanced digital competence compared to international standards. The results indicate the urgent need for comprehensive curriculum reform, faculty development, and strategic investments in advanced technology and assessment techniques to ensure that future nursing graduates are prepared to meet the demands of digital health care delivery systems. The data support the alignment of education with the provisions of Saudi Vision 2030.

## Supplementary material

10.2196/88075 Multimedia Appendix 1 Exploratory and confirmatory factor analysis results for the Canadian Nursing Informatics Competency Assessment Scale and Digital Technology Self-Efficacy scale, including factor loadings, model fit indices, and variance explained (Tables 1-6; Figures 1-3).

## Abbreviations

C-NICAS: Canadian Nursing Informatics Competency Assessment Scale
DT-SE: Digital Technology Self-Efficacy
EHR: electronic health record
ICT: information and communication technology
IMIA: International Medical Informatics Association
TIGER: Technology Informatics Guiding Education Reform
