# Supplementary material for: Psychometric Evaluation of the Canadian Nurse Informatics Competency Assessment Scale and the Digital-Technology Self-Efficacy Scale Among Saudi Nursing Students: Cross-Sectional Study
Source: JMIR Nurs. 2026 May 5;9:e88075. doi: 10.2196/88075 (PMC13143192; doi:10.2196/88075)
Supplement: Multimedia Appendix 1 [file nursing-v9-e88075-s001.docx]

**Table 1. Exploratory factor analysis and factor loadings (EFA) of C-NICAS**

| **Item number** | **Pattern matrix: factor 1** | **Pattern matrix: factor 2** | **Pattern matrix: factor 3** | **Structure matrix: factor 1** | **Structure matrix: factor 2** | **Structure matrix: factor 3** | **Communalities** | |  |
| --- | --- | --- | --- | --- | --- | --- | --- | --- | --- |
| Q1 | 0.498 | 0.265 | 0.689 | 0.830 | 0.716 | 0.906 | 0.666 | |  |
| Q2 | 0.603 | 0.350 | 0.551 | 0.902 | 0.784 | 0.880 | 0.709 | |  |
| Q3 | 0.299 | 0.922 | 0.272 | 0.673 | 0.939 | 0.599 | 0.652 | |  |
| Q4 | 0.451 | 0.686 | 0.247 | 0.760 | 0.858 | 0.675 | 0.583 | |  |
| Q5 | 0.213 | 0.839 | 0.326 | 0.658 | 0.918 | 0.697 | 0.627 | |  |
| Q6 | 0.263 | 0.725 | 0.566 | 0.661 | 0.897 | 0.827 | 0.667 | |  |
| Q7 | 0.261 | 0.606 | 0.754 | 0.707 | 0.886 | 0.948 | 0.762 | |  |
| Q8 | 0.270 | 0.204 | 0.725 | 0.689 | 0.690 | 0.985 | 0.887 | |  |
| Q9 | 0.295 | 0.663 | 0.640 | 0.610 | 0.638 | 0.709 | 0.815 | |  |
| Q10 | 0.406 | 0.925 | 0.424 | 0.548 | 0.934 | 0.713 | 0.675 | |  |
| Q11 | 0.323 | 0.746 | 0.593 | 0.630 | 0.899 | 0.831 | 0.677 | |  |
| Q12 | 0.366 | 0.821 | 0.491 | 0.569 | 0.891 | 0.745 | 0.627 | |  |
| Q13 | 0.214 | 0.749 | 0.330 | 0.425 | 0.667 | 0.375 | | 0.331 | |
| Q14 | 0.683 | 0.655 | 0.426 | 0.810 | 0.805 | 0.523 | | 0.596 | |
| Q15 | 0.670 | 0.223 | 0.517 | 0.848 | 0.630 | 0.788 | | 0.582 | |
| Q16 | 0.513 | 0.367 | 0.518 | 0.801 | 0.729 | 0.802 | | 0.568 | |
| Q17 | 0.636 | 0.668 | 0.343 | 0.821 | 0.839 | 0.586 | | 0.615 | |
| Q18 | 0.329 | 0.206 | 0.881 | 0.736 | 0.657 | 0.956 | | 0.681 | |
| Q19 | 0.498 | 0.619 | 0.263 | 0.702 | 0.755 | 0.555 | | 0.460 | |
| Q20 | 0.585 | 0.481 | 0.933 | 0.864 | 0.559 | 0.905 | | 0.844 | |
| Q21 | 0.991 | 0.424 | 0.388 | 0.808 | 0.775 | 0.616 | | 0.790 | |
| Q22 | 0.887 | 0.227 | 0.280 | 0.951 | 0.671 | 0.709 | | 0.670 | |
| Q23 | 0.430 | 0.611 | 0.754 | 0.992 | 0.639 | 0.640 | | 0.729 | |
| Q24 | 0.957 | 0.260 | 0.269 | 0.964 | 0.617 | 0.691 | | 0.687 | |
| Q25 | 0.511 | 0.227 | 0.247 | 0.967 | 0.615 | 0.626 | | 0.691 | |
| Q26 | 0.740 | 0.234 | 0.471 | 0.883 | 0.633 | 0.776 | | 0.610 | |
|  |  | **Kaiser–Meyer–Olkin measure of sampling adequacy = 0.976, indicating excellent suitability of the data for factor analysis.** | | | | | | | |

**Table 2. Confirmatory Factor Analysis (CFA) for C-NICAS**

| **Model** | **χ²** | **df** | ***P*** | **CFI** | **IFI** | **RMSEA (90% CI)** |
| --- | --- | --- | --- | --- | --- | --- |
| **C‑NICAS 4‑factor** | **2.02** | **1** | **.001** | **1.000** | **1.000** | **0.081** |

*Model fit parameters: CFI=1.000, IFI=1.000, RMSEA=0.081. CFI = comparative fit index; IFI = incremental fit index; RMSEA = root mean square error of approximation. The model showed acceptable fit with χ²/df=2.02, P=.001.*

**Table 3. Confirmatory factor analysis for DT-SE**

| **Test** | **Value** |
| --- | --- |
| **Kaiser–Meyer–Olkin measure of sampling adequacy** | **0.930** |
| **Bartlett’s test of sphericity: χ² (df), *P*** | **2987.109 (136), *P*<.001** |

**Table 4. Total variance explained of DT-SE**

| **Component** | **Initial eigenvalues: total** | **% of variance** | **Cumulative %** |
| --- | --- | --- | --- |
| **1** | **8.156** | **47.975** | **47.975** |
| **2** | **2.156** | **12.682** | **60.657** |
| **3** | **1.200** | **7.061** | **67.717** |

**Table 5. Rotated factor loadings of DT-SE**

| **Item** | **Factor 1** | **Factor 2** | **Factor 3** |
| --- | --- | --- | --- |
| DT15 | 0.788 | – | – |
| DT13 | 0.787 | – | – |
| DT14 | 0.708 | – | – |
| DT12 | 0.704 | – | – |
| DT9 | 0.691 | – | – |
| DT10 | 0.689 | – | – |
| DT17 | 0.599 | – | – |
| DT4 | – | 0.679 | – |
| DT3 | – | 0.673 | – |
| DT2 | – | 0.669 | – |
| DT5 | – | 0.620 | – |
| DT6 | – | 0.527 | – |
| DT8 | – | – | 0.810 |
| DT11 | – | – | 0.739 |
| DT7 | – | – | 0.727 |
| DT16 | – | – | 0.587 |
| DT1 | – | – | 0.560 |

**Table 6. Confirmatory factor analysis for DT-SE**

| **Indicator** | **Value** |
| --- | --- |
| Sample size | 243 |
| Rows with missing | 20 |
| −2 log likelihood | 11290.367 |
| Iterations | 11 |
| Number of parameters | 51 |
| AICc | 11413.841 |
| BICu | 198.75003 |
| Chi-square | 877.10282 |
| df | 119 |
| *P* | <.001 |
| CFI | 0.755 |
| RMSEA | 0.146 |
| Lower 90% CI (RMSEA) | 0.137 |
| Upper 90% CI (RMSEA) | 0.155 |
|  |  |


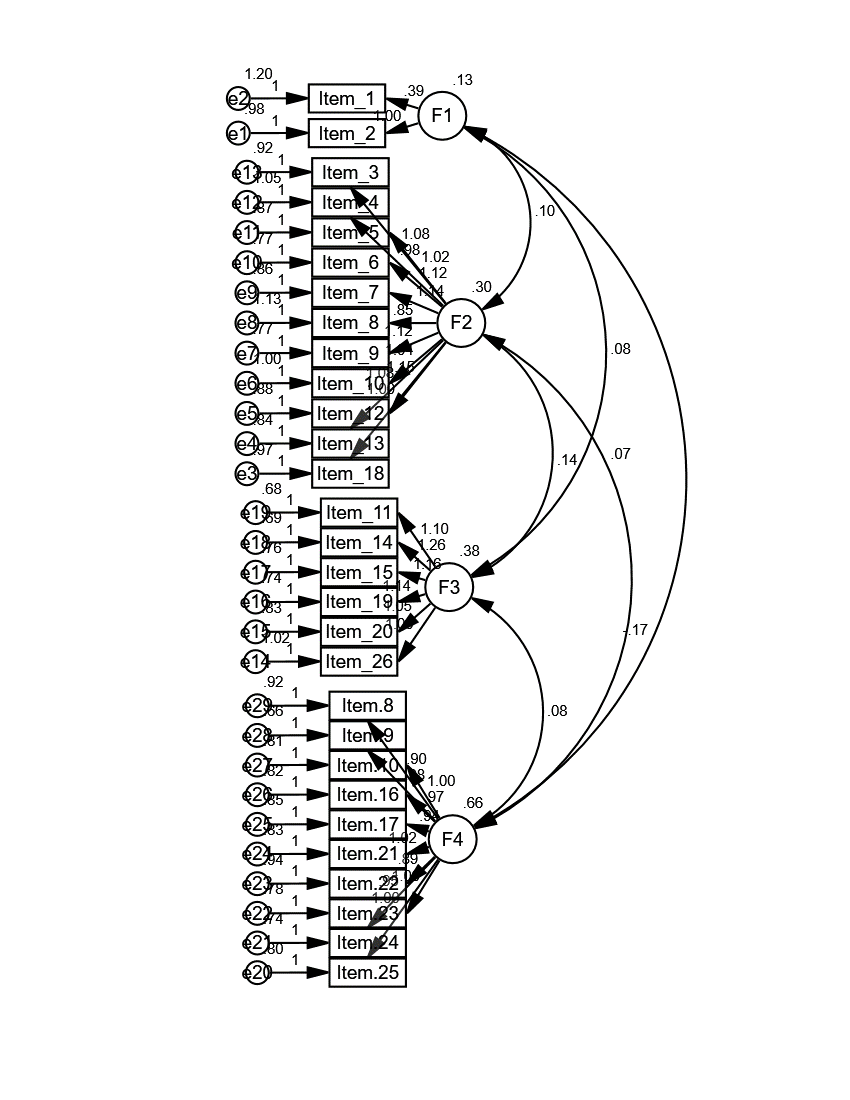


**Figure 1. Exploratory factor analysis and factor loadings**


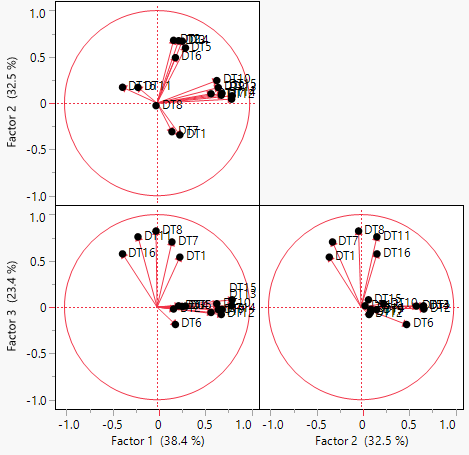


**Figure 2. Factor loadings plot of DT-SE**


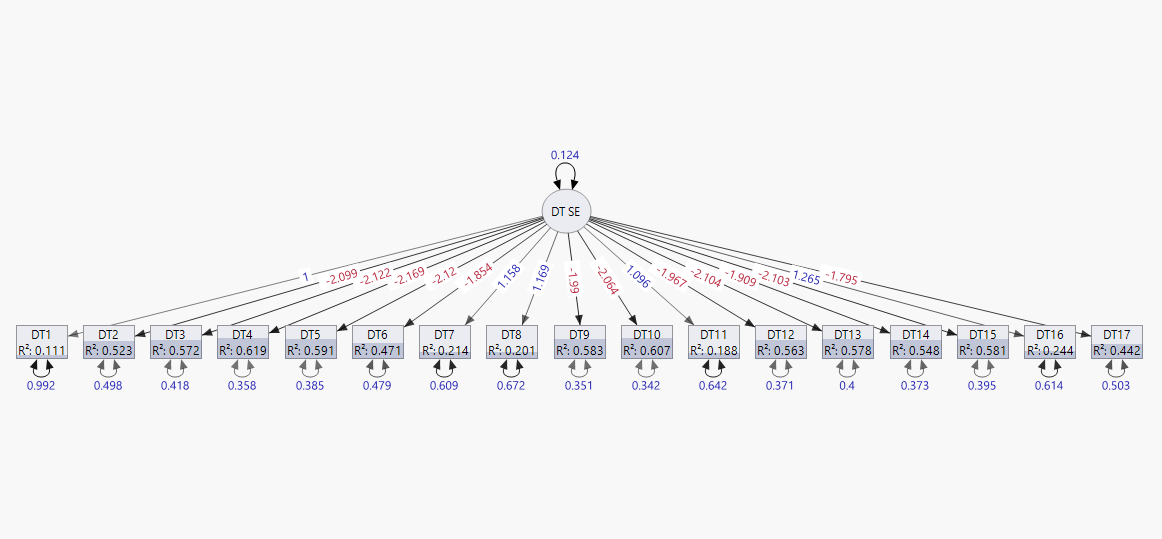


**Figure 3. Confirmatory factor analysis for DT-SE**
